# A case report of primary orbital non-Hodgkin’s lymphoma causing complete vision loss

**DOI:** 10.3205/oc000043

**Published:** 2016-04-15

**Authors:** Neerav Lamba, Douglas P. Dworak, Shyam A. Patel, Rohini Chennuri

**Affiliations:** 1 John H. Stroger, Jr. Hospital of Cook County, Division of Ophthalmology, Chicago, USA; 2 John H. Stroger, Jr. Hospital of Cook County, Division of Pathology, Chicago, USA

**Keywords:** Non-Hodgkin’s, NHL, lymphoma, primary orbital lymphoma, orbital

## Abstract

A 29-year-old male with acquired immunodeficiency syndrome presented with a week of left eye blurriness, which then progressed to complete vision loss. On exam, the left pupil was nonreactive to light, and fundoscopy showed significant optic nerve edema. CT and MRI of the left orbit showed a mass lesion compressing the posterior aspect of the sclera with diffuse thickening and contrast enhancement of the retrobulbar portion of the left optic nerve. The lesion demonstrated low T1 and intermediate T2 intensities and heterogeneous contrast enhancement and measured 17.4 mm x 15 mm x 10.6 mm. Anterior orbitotomy with exploration and biopsy were performed. Immunohistochemical studies confirmed diffuse large B-cell lymphoma and a workup showed no systemic involvement. Plans for treatment with chemotherapy and radiation were initiated.

Even though rare, primary orbital NHL should be in the differential for relatively acute blindness without other symptoms, especially in patients with AIDS.

## Background

Primary orbital lymphoma is a rare condition and site for non-Hodgkin’s lymphoma. The most common type of NHL is diffuse large B-cell lymphoma. NHL can have extra-nodal presentation in 25% to 35% of patients. However, extra-nodal lymphoma of the head is very rare [1]. Even though rare in the head and neck, lymphoma is thought to be the most common orbital malignancy, with its incidence increasing [2]. Of these, MALT tumors are the most common, followed by DLBCL [3]. 

Non-Hodgkin’s lymphoma is a heterogeneous group of malignant lymphomas that reflect the development stages of lymphocytes, with the majority arising from a B-cell origin. Even though primary orbital lymphomas represent 1% of NHLs and 8% of extra-nodal NHLs, POLs make up about half of adult primary orbital malignancies [4], [5]. Unlike most primary orbital NHLs, our patient was diagnosed with POL at the young age of 29 years. This was likely contributed by the patient’s poor HIV control and low CD4 count (AIDS). 

The mean duration of symptoms of a POL at presentation is approximately 18 months (range: 10 days to 10 years), which is significantly greater than our patient’s duration of one week [6]. Tatsugawa et al. described a similar case of orbital lymphoma causing blindness without other symptoms in a 71-year-old patient; however, visual acuity deteriorated for 3 months in the left eye and 1 month in the right eye until there was no light perception bilaterally [7]. Less than 20% of patients present with any kind of visual acuity loss, and complete vision loss is rare, especially without other common symptoms. Tranfa et al. showed that there are 8 signs and symptoms that are more prevalent than vision loss for a POL, with the most common being a palpable mass [6].

CT scans generally shows a well-defined, homogenous, high-density mass that is nodular and has distinct margins [8]. MRI usually shows hypointensity particularly on T1-weighted images and homogenous enhancement [9]. Most orbital lymphoid tumors tend to conform to structures around them without bony erosions. The MRI images of our patient do agree with some of these findings such as hypointensity on T1-weighted imaging; however, our patient’s lesion did show heterogeneous enhancement. Another rare feature is the intraconal position. Priego et al. estimated that 10.5% of orbital lymphomas have only intraconal involvement [10]. 

Diagnosis of POLs is accomplished by tissue studies and imaging. Standard treatment for DLBCL includes R-CHOP with or without radiation. R-CHOP is safe for AIDS patients and can be combined with HAART therapy. 

It is rare for the presenting symptom of a POL to be complete blindness without other symptoms of mass effect. We describe a case of a primary orbital NHL causing complete blindness in the left eye of a 29-year-old male with AIDS after a week of blurriness. 

## Case report

**Figure 1:**
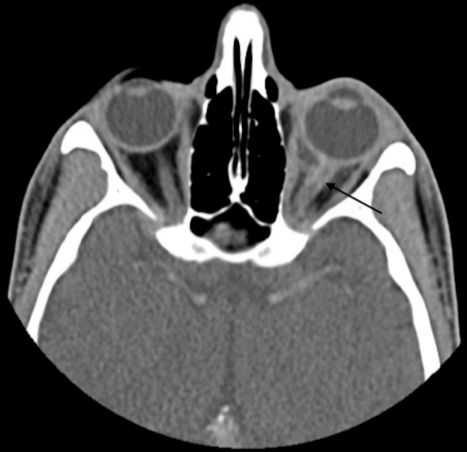
Computed tomography showing a retrobulbar orbital mass in the left orbit (black arrow)

**Figure 2:**
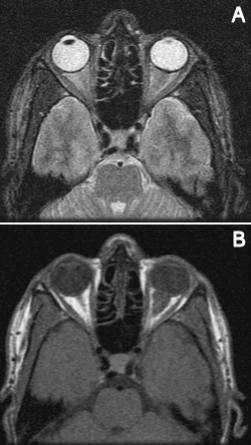
Magnetic resonance imaging showing a retrobulbar orbital mass in the left orbit

A 29-year-old Hispanic male presented with a week of blurriness in his left eye that progressed to complete vision loss. He was HIV positive for 6 years with a latest CD4 count of 100 and a viral load of 617 copies/mL on HAART therapy. He noted fevers and chills, but no other symptoms. On examination, the left pupil was fixed with no response to light. A reverse relative afferent pupillary defect was present in the right eye. The left optic nerve was edematous with blurring of margins. A CT scan showed enhancement of the retrobulbar optic nerve of the left eye with an adjacent, discrete soft tissue enhancement bordering the posterior globe (Figure 1 (Fig. 1)). Two weeks later, an MRI showed diffuse thickening and contrast enhancement involving the retrobulbar portion of the left optic nerve associated with a surrounding soft tissue lesion within the intraconal orbital fat, and the lesion seemed to be extending more posteriorly than the previous CT image (Figure 2 (Fig. 2)). The mass abutted the posterior aspect of the sclera. The lesion demonstrated low T1 and intermediate T2 signal intensities and heterogeneous contrast enhancement. There was also nodular contrast enhancement of the left optic nerve disc. The size of the lesion measured 17.4 mm x 15 mm x 10.6 mm. Cerebrospinal fluid studies were unremarkable. An anterior orbitotomy with exploration and biopsy of the left orbital mass was performed.

**Figure 3:**
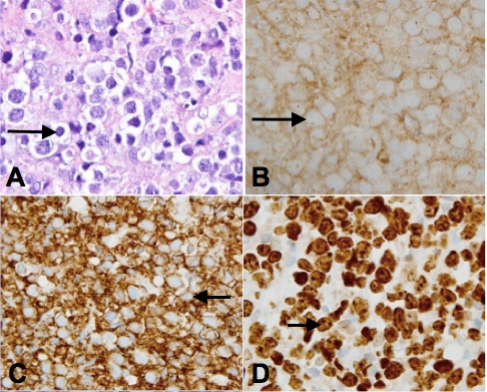
H&E section of the tumor shows a diffuse growth pattern of large, dysplastic lymphocytes with vesicular nuclei (A), which are positive for CD10 (B) and CD20 immunostain (C). The Ki67 (D) immunostain demonstrates a high (>90% of tumor cells) proliferative index.

Histology of the mass showed tissue diffusely infiltrated with large, dysplastic lymphocytes with vesicular nuclei (Figure 3A (Fig. 3)). Immunohistochemical stains showed that the lymphoid cells were positive for CD10 and CD20 (Figure 3B and 3C (Fig. 3)) and were negative for CD3 and keratin AE1/AE3. Ki-67 demonstrated proliferative activity in greater than 90% of tumor cells (Figure 3D (Fig. 3)). Additional immunohistochemical stains showed that the tumor was positive for BCL-2 and BCL-6. The patient was diagnosed with DLBCL (germinal center type) and was referred to oncology. He underwent staging and there was no other evidence of the disease in any other system in the body. He was started on R-CHOP chemotherapy. He was given one round of R-CHOP before he was unfortunately lost to follow-up; one round of R-CHOP administration did not improve his visual acuity.

## Conclusions

Even though rare, primary orbital NHL should be in the differential for subacute blindness especially in patients with AIDS. After imaging and diagnostic biopsy, patients should be immediately staged and treated. For DLBCL, the standard treatment includes R-CHOP with HAART for AIDS patients.

## Abbreviations

BCL: B-cell lymphoma

CD: cluster of differentiation

CT: computerized axial tomography

DLBCL: diffuse large B-cell lymphoma

HAART: highly active antiretroviral therapy

MALT: mucosa associated lymphoid tissue

MRI: magnetic resonance imaging

NHL: non-Hodgkin’s lymphoma

POL: primary orbital lymphoma

R-CHOP: rituximab, cyclophosphamide, doxorubicin, vincristine, prednisone

## Notes

### Ethics

Informed consent was obtained from the patient, and this report is HIPAA-compliant.

### Competing interests

The authors declare that they have no competing interests.

## References

[1] Newton R, Ferlay J, Beral V, Devesa SS (1997). The epidemiology of non-Hodgkin’s lymphoma: comparison of nodal and extra-nodal sites. Int J Cancer.

[2] Margo CE, Mulla ZD (1998). Malignant tumors of the orbit. Analysis of the Florida Cancer Registry. Ophthalmology.

[3] Rey-Porca C, Pérez-Encinas M, González F (2008). Linfomas orbitarios. Presentación de nueve casos. Arch Soc Esp Oftalmol.

[4] Freeman C, Berg JW, Cutler SJ (1972). Occurrence and prognosis of extranodal lymphomas. Cancer.

[5] Volpe NJ, Gausas RE (1999). Optic nerve and orbital tumors. Neurosurg Clin N Am.

[6] Tranfa F, Di Matteo G, Strianese D, Forte R, Bonavolontà G (2001). Primary orbital lymphoma. Orbit.

[7] Tatsugawa M, Noma H, Mimura T, Funatsu H (2009). Unusual orbital lymphoma undetectable by magnetic resonance imaging: a case report. J Med Case Rep.

[8] Flanders AE, Espinosa GA, Markiewicz DA, Howell DD (1987). Orbital lymphoma. Role of CT and MRI. Radiol Clin North Am.

[9] Jin CW, Rana N, Wang Y, Ma S, Li M, Zhang M (2010). CT and MRI features of Primary Orbital Lymphoma: review of 14 cases. Asian J Med Sci.

[10] Priego G, Majos C, Climent F, Muntane A (2012). Orbital lymphoma: imaging features and differential diagnosis. Insights Imaging.

